# Factors Associated with Worse Outcome in Early Stage Gastric Cancer Using the Surveillance, Epidemiology, and End Results (SEER) Database

**DOI:** 10.7759/cureus.7360

**Published:** 2020-03-22

**Authors:** Yanal Alnimer, Ayman Qasrawi

**Affiliations:** 1 Internal Medicine, Riverside Health System, Glen Allen, USA; 2 Hematology and Medical Oncology, University of Kentucky College of Medicine, Lexington, USA

**Keywords:** early gastric cancer, stage 1b gastric cancer, adjuvant chemotherapy

## Abstract

Background

The benefit of adjuvant treatment in gastric adenocarcinoma patients with involvement of the muscularis propria but not beyond is unclear. We aim to establish a model that identifies the factors that adversely affect the prognosis in these patients.

Methods

We used the Surveillance, Epidemiology, and End Results (SEER) database to identify subjects with stage T2aN0M0 gastric adenocarcinoma who had tumor resection between 2004 and 2015. Data pertaining to the following variables were collected: age, gender, ethnicity, tumor size, grade, site, number of lymph nodes (LNs) being examined, and extent of surgery.

Results

A total of 1307 patients met our inclusion criteria. The five-year overall survival (OS) was 65%. The following factors were significantly associated with a shorter OS in univariate analysis. Age > 60 years, non-Hispanic whites and non-Hispanic blacks, patients with less than 15 lymph nodes examined at the time of surgery, tumors at the fundus and cardia of the stomach, and those who underwent endoscopic resection or had partial esophagectomy. On multivariate Cox regression, the following factors were predictors for worse OS: age > 60 years with a hazards ratio (HR) = 2.03 (95% CI: 1.49-2.76), patients with less than 15 lymph nodes examined with HR = 1.72 (95% CI: 1.34-2.20), non-Hispanic whites and non-Hispanic blacks with HR = 1.62 (95% CI: 1.26-2.08), and tumors within the cardia and fundus of the stomach with HR = 1.51 (95% CI: 1.21-1.89).

Conclusion

Patients with stage T2aN0M0 gastric cancer who had their tumor located at the cardia or fundus of the stomach or those with inadequate lymph nodes resection had inferior survival and could potentially benefit from adjuvant chemotherapy.

## Introduction

Introduction

Gastric cancer is one of the leading causes of cancer-related deaths worldwide [1]. Early gastric cancers (EGC) are usually asymptomatic and thus discovered incidentally during endoscopic procedures. Additionally, those patients have much better outcomes as compared to those with a symptomatic advanced-stage disease [2]. Five-year survival ranges from 96% in those with EGC to 9% with a locally advanced non-metastatic disease [3].

In the United States, the five-year survival for stage IA gastric cancer is 71%. In comparison, survival for stage IB falls dramatically to 57% [3]. Patients with stage IB gastric cancer include two categories: patients with their disease limited to the submucosa with the involvement of one to two regional lymph nodes and those with muscularis propria but without the involvement of regional lymph nodes. While surgical resection followed by adjuvant chemotherapy is clearly defined as a treatment modality for the former, the effect of adjuvant chemotherapy for the latter is debatable [4]. No randomized controlled trials (RCTs) were designed to determine the outcome of adjuvant chemotherapy, radiation, or both in those subjects. The Intergroup (0116) trial showed a clear benefit from adjuvant chemoradiation when compared to surgery alone in surgically resected gastric cancer [5]. Although this trial included subjects whose cancer had invaded the muscularis propria, no distinction was made between those whose cancer had invaded muscularis propria only and those with subserosa involvement. Therefore, conclusions cannot be made in those whose cancer is limited to the muscularis propria.

In our study, we aimed to identify the high-risk features that could predict worse outcomes in patients who had gastric cancer that was limited to the muscularis propria using the Surveillance, Epidemiology, and End Results Program (SEER database).

## Materials and methods

Patients

We used the SEER database to identify subjects with gastric adenocarcinoma, which was limited to the muscularis propria, and subsequently underwent resection. Since the seventh Tumor, Node, Metastasis (TNM ) staging system was implemented at the SEER database after 2015, we decided to use the sixth TNM staging system in order to include the largest possible number of patients. We used the SEER database named “SEER 18 Regs Custom Data (with additional treatment fields). Nov 2017 Sub (2000-2015) <Katrina/Rita Population Adjustment>” to identify the five-year overall survival (OS) in patients who had TNM stage T2aN0M0 gastric adenocarcinoma according to the sixth TNM staging system (patients with muscularis propria involvement but not beyond) between 2004-2015. The following International Classification of Diseases for Oncology (ICD-O-3) codes were included: 8140, 8142, 8144, 8145, 8210, 8211, 8255, 8260, 8261, 8263, 8480, 8481, and 8490. The codes C16.0 to C16.9 were used to identify the labeled tumor’s primary site as within the stomach.

The following variables were collected at the time of tumor removal, whether surgically or endoscopically: patient’s age, gender, year of diagnosis, tumor size, tumor anatomic site, histology and grade, number of lymph nodes examined, and extent of surgical lymph nodes dissection. A total number of 1307 of patients were identified that met the inclusion criteria and included in the study.

Statistical analysis

Data were presented descriptively as proportions, means, or medians as appropriate. The primary endpoint of the study was overall survival (OS) at 60 months after diagnosis. Patients who were alive at the time of the last follow-up were censored at that time. Survival was estimated by the Kaplan-Meier method and comparisons were made by the log-rank test. All factors with p-values <0.1 in univariate analysis were entered in a multivariate stepwise Cox proportional hazard ratio model. All statistical analyses were performed using the MedCalc ® 18.11.6 software (MedCalc Software Ltd., Ostend, Belgium).

## Results

Baseline characteristics

The median age was 69-years-old (range 30-97). In our cohort, the tumor size ranged from 2 mm to 300 mm with a median of 30 mm. Thirty-six percent of the patients were females and 29% (n = 383) had their tumor located in the cardia and fundus of the stomach. The histologic subtypes were classified into five groups: diffuse, adenocarcinoma not otherwise specified (NOS), intestinal, signet ring, and mucinous. Other rare subtypes, including adenocarcinoma with mixed subtypes (n = 30), adenocarcinoma arising from a polyp or an adenoma (n = 21), tubular adenocarcinoma (n = 17), and papillary adenocarcinoma (n = 5), were grouped with adenocarcinoma NOS to simplify statistical analysis. Adenocarcinoma NOS and intestinal type were the predominant histological subtypes in our cohort and comprised 60% (n = 788) and 18% (n = 238) of the patients, respectively. The main characteristics of our patients’ cohort are shown in Table 1.

**Table 1:** Baseline characteristics of all patients who met the inclusion criteria (1307) NOS: not otherwise specified; LNs: lymph nodes

Variable	Number (Percentage)
No.	1307 (100%)
Median Age (Range)	69 years (30-97)
Age Group	
60 years or older	993 (76%)
< 60 years	314 (24%)
Sex	
Male	839 (64%)
Female	468 (36%)
Race	
Hispanic	235 (18%)
Non-Hispanic white	625 (48%)
Non-Hispanic Asian or Pacific Islander	273 (21%)
Non-Hispanic black	161 (12%)
Non-Hispanic American Indian/Alaskan native	12 (0.9%)
Non-Hispanic unknown	1 (0.1%)
Median Tumor Size (Range)	30 mm (2-300)
Tumor Grade	
Well or moderately differentiated	579 (44%)
Poorly differentiated	674 (52%)
Unknown	54 (4%)
Tumor Location	
Cardia and fundus body	383 (29%)
Greater and lesser curvatures	354 (27%)
Antrum and pylorus	424 (32%)
Unknown	146 (11%)
Histological Subtype	
Adenocarcinoma	788 (60%)
NOS	238 (18%)
Intestinal type	181 (14%)
Signet ring	60 (5%)
Diffuse mucinous	40 (3%)
Type of Resection	
Endoscopic or local resection	32 (2%)
Partial or total gastrectomy	970 (74%)
Partial esophagectomy during gastric tumor resection	295 (23%)
Unknown	10 (1%)
Number of Regional LNs Examined	
< 15	803 (61%)
> 15	495 (38%)
Unknown	9 (1%)
Patients Who Received Chemotherapy	
No/Unknown	893 (68%)
Yes	414 (32%)

Survival analysis

**Figure 1:**
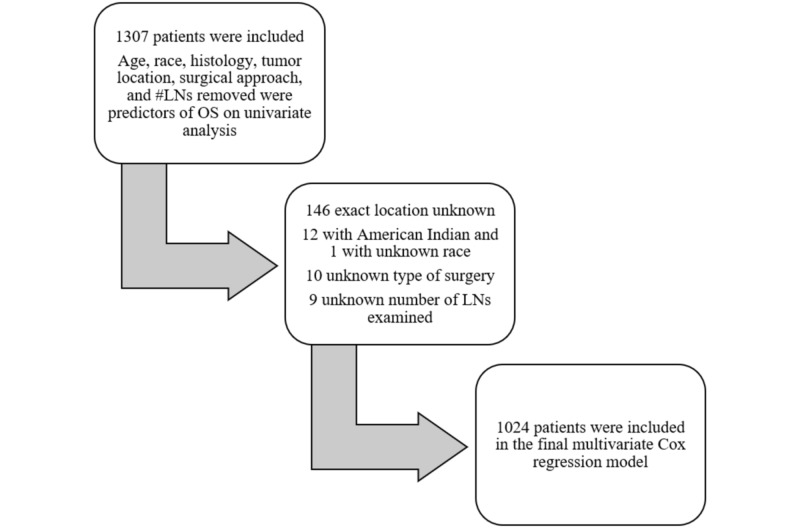
Schematic showing the details of the patients included in the multivariate Cox regression model LNs: lymph nodes

**Figure 2:**
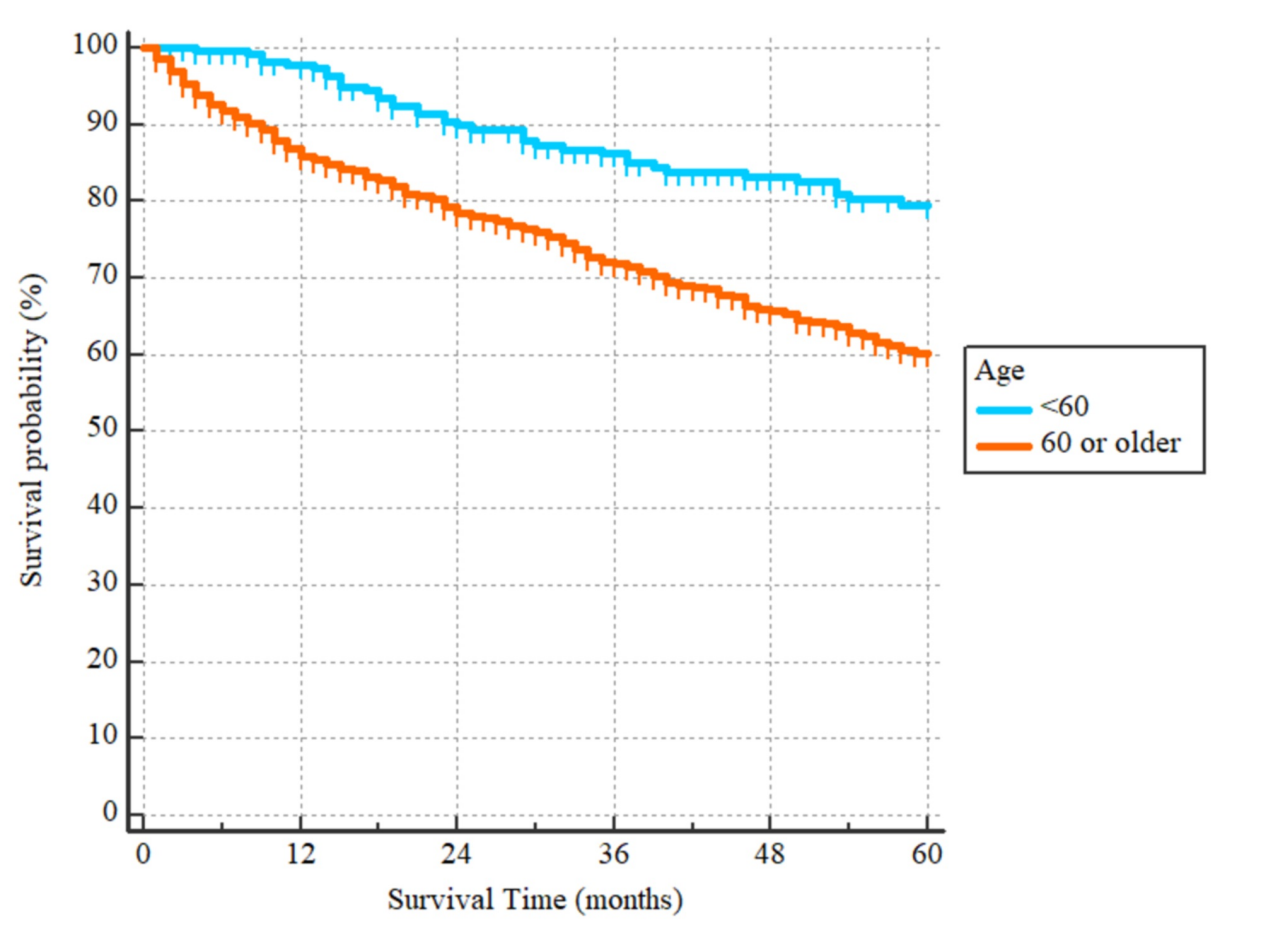
Overall survival curves according to the patients’ age HR = 1.95 with 95% CI = 1.50 to 2.53, p < 0.0001

**Figure 3:**
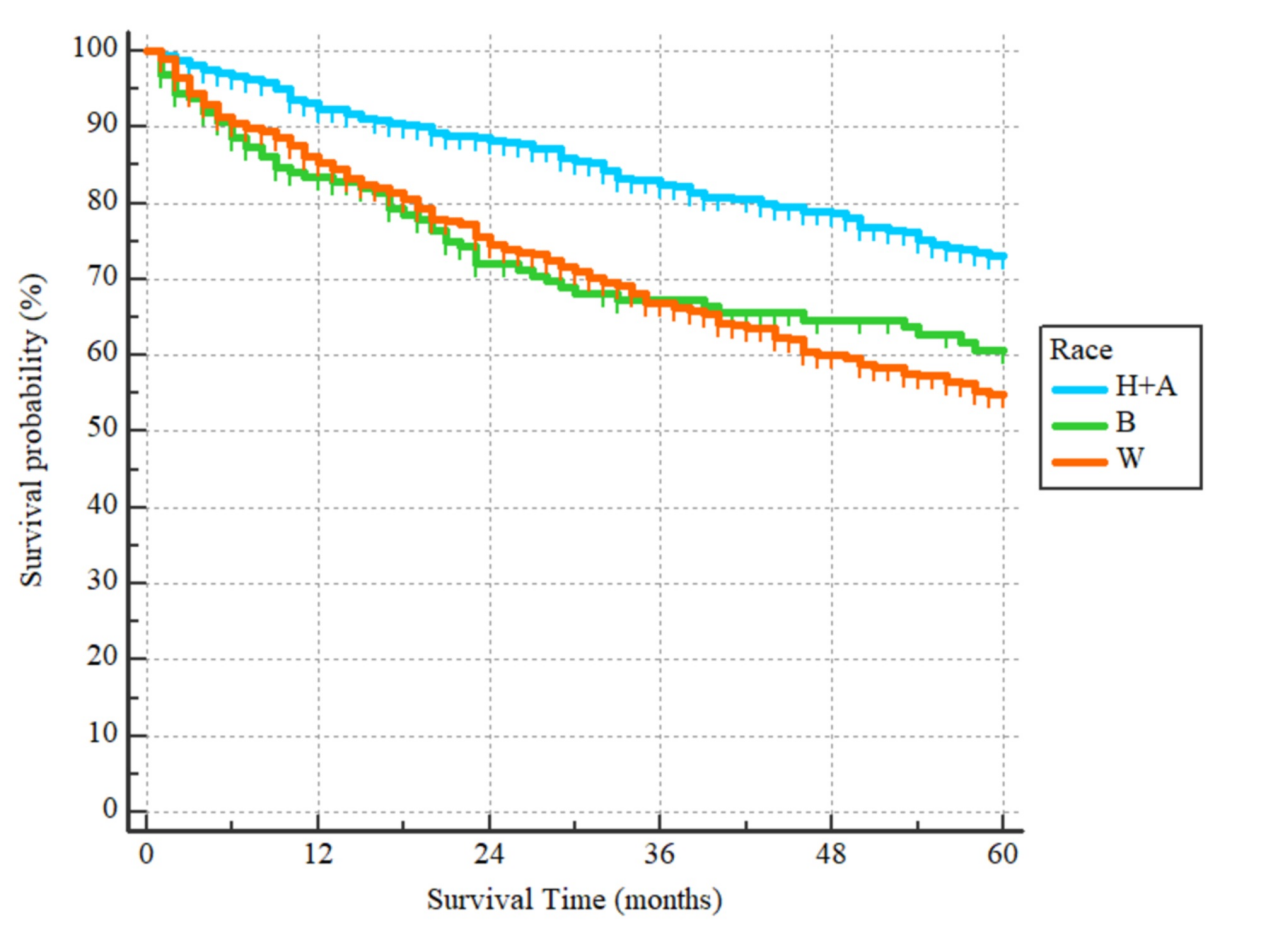
Overall survival curves according to the patients’ race H+A: combined Hispanics (All Races) and non-Hispanic Asians or Pacific Islanders. B: non-Hispanic blacks. W: non-Hispanic whites. There were only 12 patients who were American Indian/Alaska natives and an additional patient with an unknown race and were excluded from our analysis. Hazard ratios: W/H+A = 1.99 (95% CI = 1.61-2.44), B/H+A = 1.81 (95% CI = 1.31-2.50), p < 0.0001

**Figure 4:**
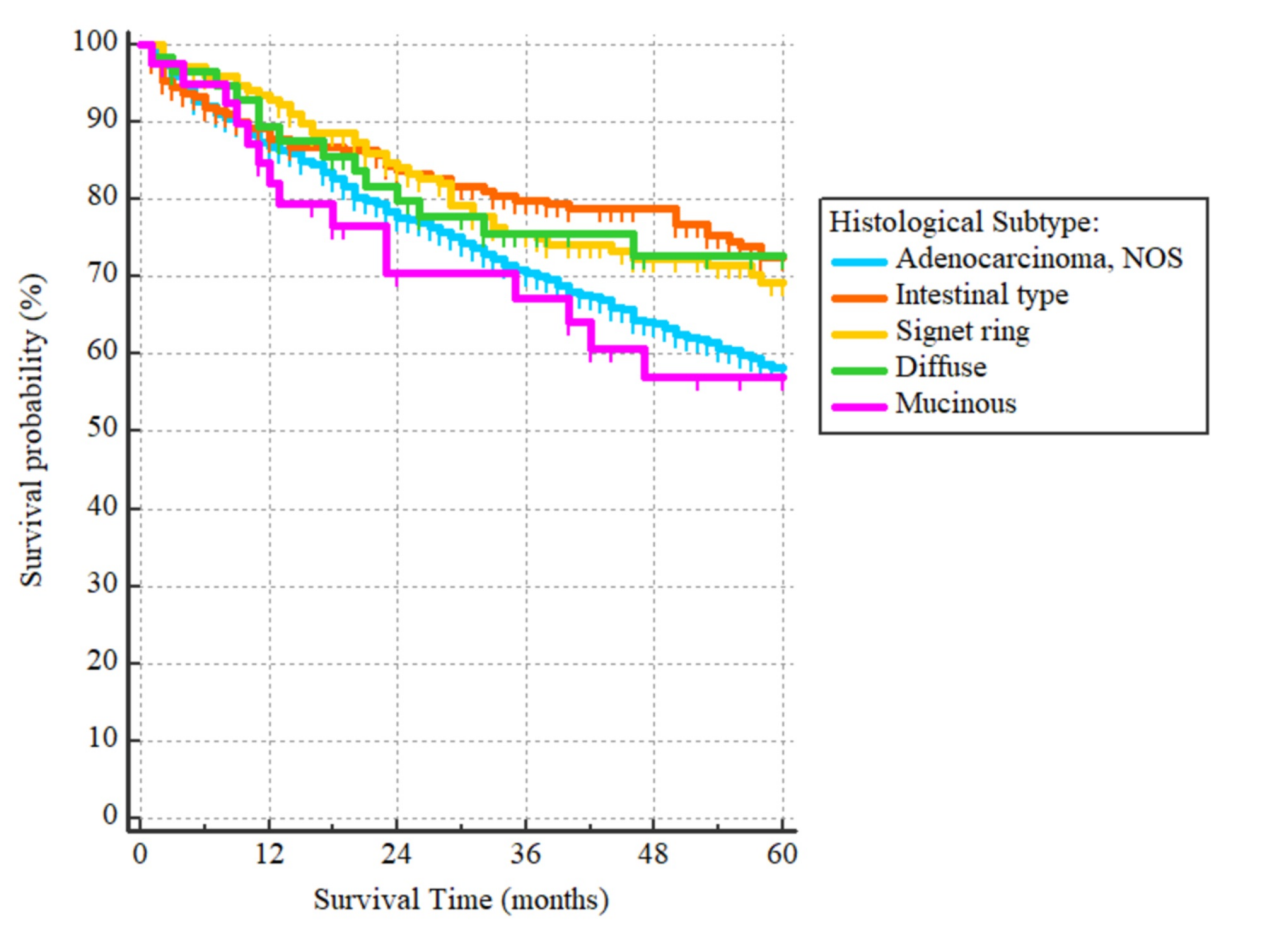
Overall survival curves according to the histological subtypes The OS was significantly worse for adenocarcinoma, NOS in comparison to intestinal (hazard ratio = 1.59 (95% CI: 1.22-2.06)) and signet ring subtypes (hazard ratio = 1.44, (95% CI: 1.08-1.92)). Other hazard ratios were not significant.

After a median follow-up period of 43 months (range 1 to 60) for the 1024 patients included in the survival analysis, there were 297 events. The three-year and five-year OS were 75% and 65%, respectively, with the median being unreached. On univariate analysis, patients who were 60 years or older had worse outcomes as compared to those younger than 60, with HR = 1.86, (95% CI 1.50-2.33), p < 0.0001 (Figure 1). Non-Hispanic whites and non-Hispanic blacks had worse OS as compared to Hispanics (all races) and non-Hispanic Asians/Pacific Islanders with HR = 1.99 (95% CI: 1.61-2.44) for the non-Hispanics whites and HR = 1.81 (95% CI: 1.31-2.50) for non-Hispanic blacks, p < 0.0001 (Figure 2). With regards to histological subtypes, the OS was significantly worse for adenocarcinoma NOS in comparison to the intestinal (HR = 1.59 (95% CI: 1.22-2.06)) and signet ring subtypes (HR = 1.44, (95% CI: 1.08-1.92)). The p-value for the whole comparison was 0.0043. The Kaplan-Meier curves are demonstrated in Figure 3. With respect to the tumor’s primary site, tumors located within the cardia and fundus of the stomach had worse outcomes as compared to those located in the antrum/pylorus or body with HRs of 1.73 (95% CI: 1.34-2.23) and 1.66 (95% CI 1.27-2.16), respectively, p < 0.0001, as demonstrated by Figure 4.

**Figure 5:**
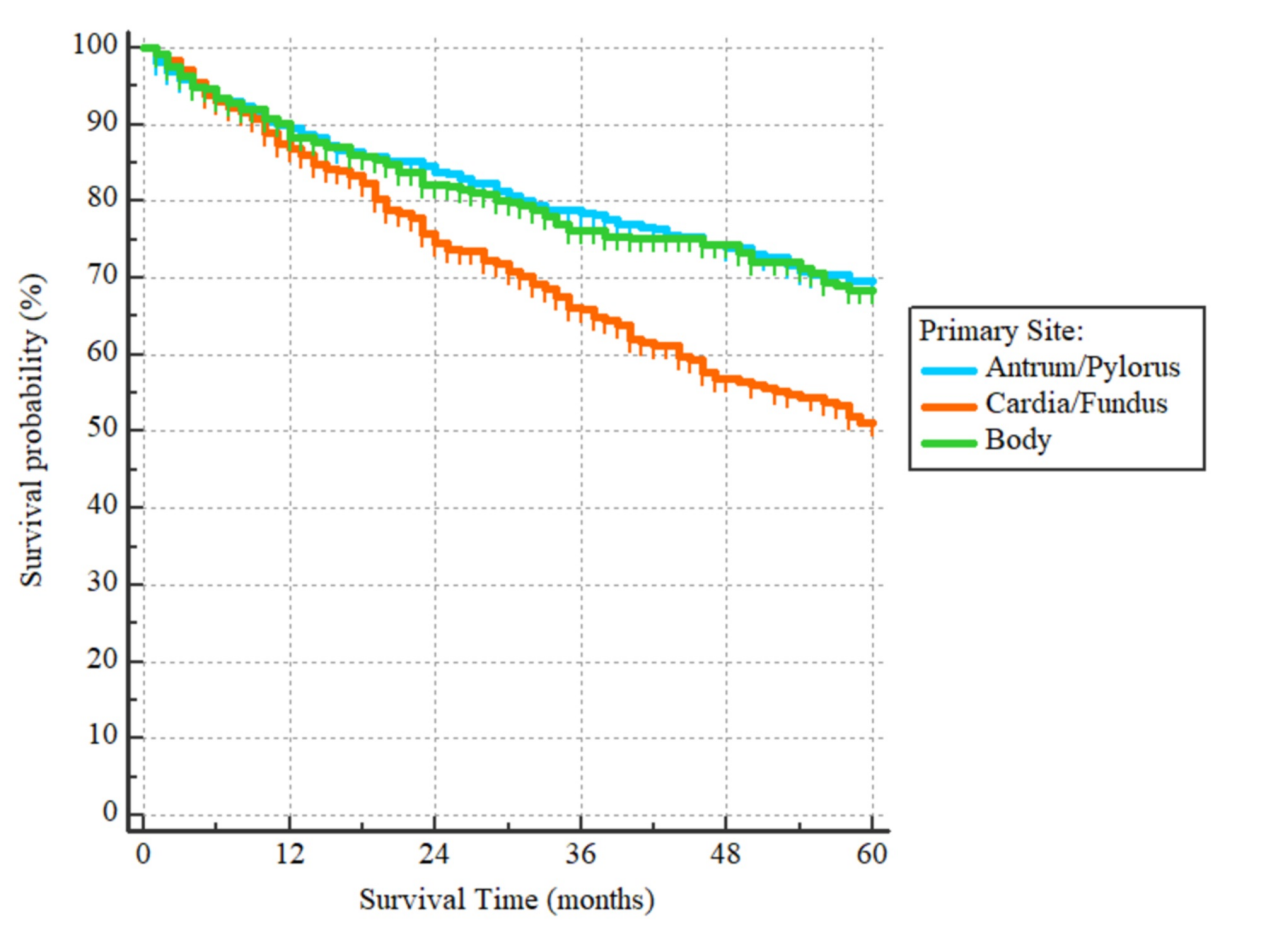
Overall survival curves according to the location of the primary tumor Hazard ratios for tumors located in the cardia/fundus compared to those located in the antrum/pylorus or body are 1.73 (95% CI: 1.34-2.23) and 1.66 (95% CI 1.27-2.16), respectively, p < 0.0001.

**Figure 6:**
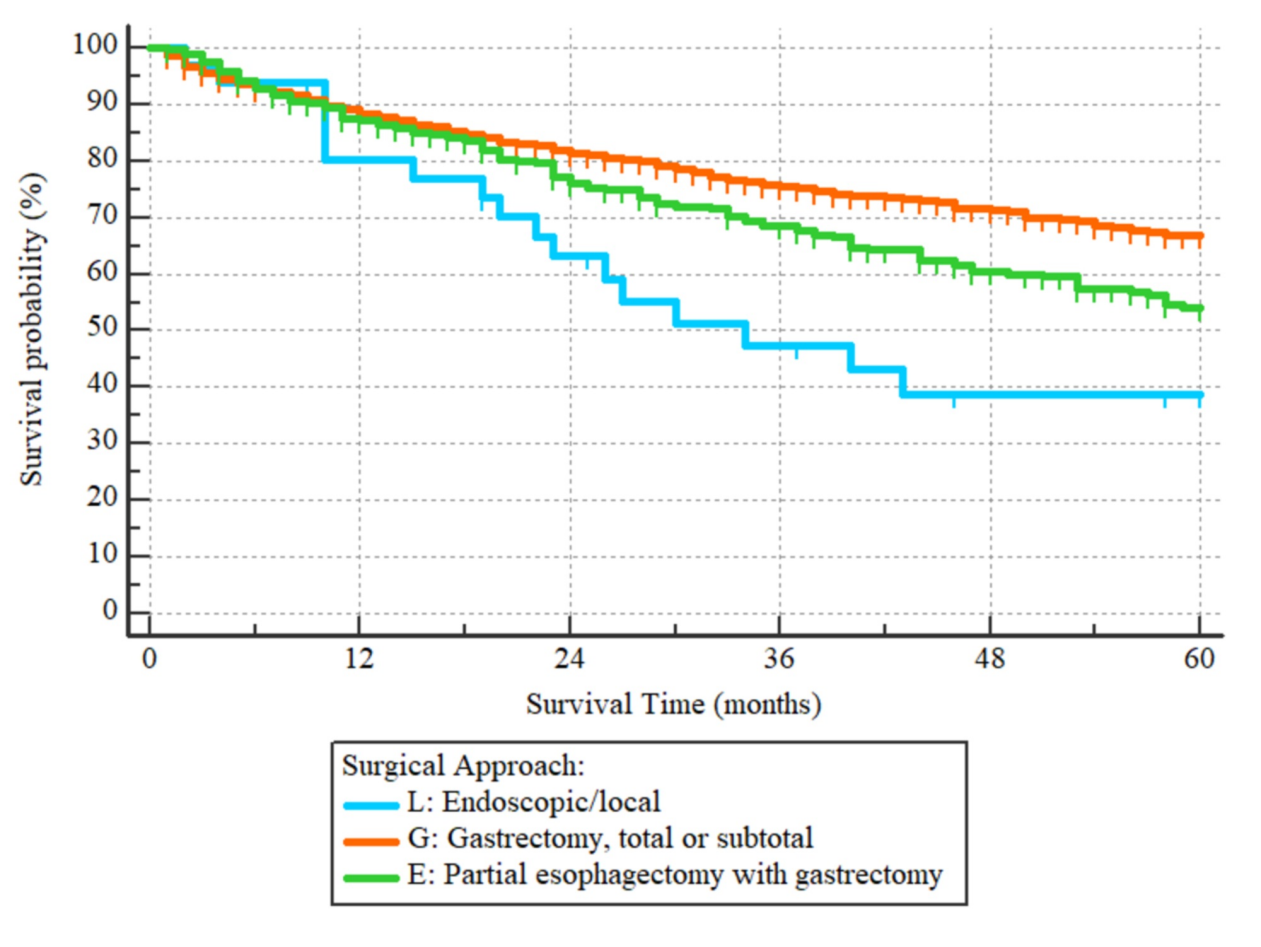
Overall survival curves according to the surgical approach L: endoscopic/local resection. G: partial or total gastrectomy. E: patients who had partial esophagectomy during gastric tumor resection Hazard ratios: L/G = 2.27 (95% CI: 1.14-4.50), L/E = 1.59 (95% CI: 0.79-3.22), E/G = 1.42 (95% CI: 1.12-1.80). p = 0.0001

**Figure 7:**
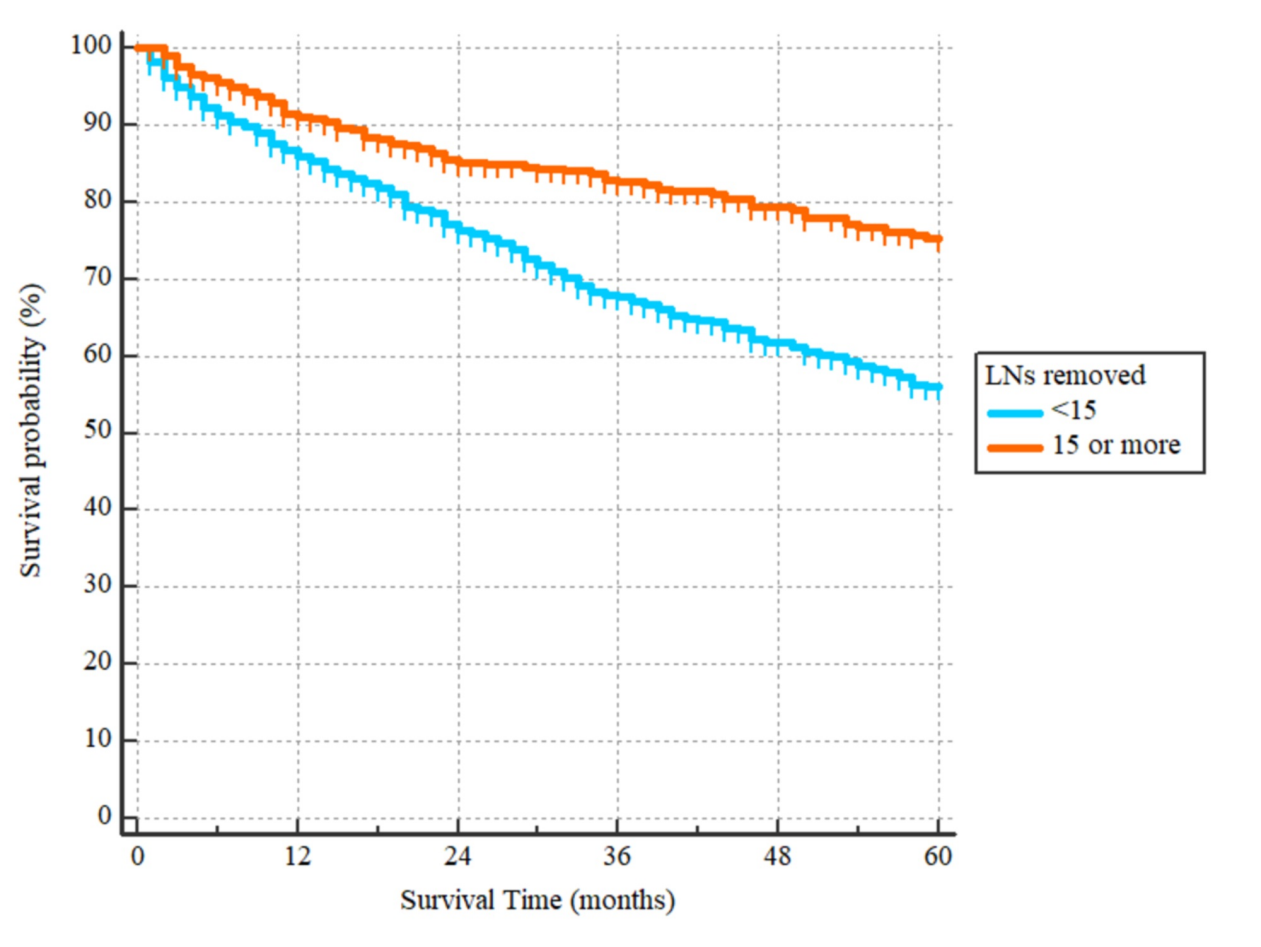
Overall survival curves according to degree of lymphadenectomy Patients who had <15 LNs removed had worse outcomes with a hazard ratio of 1.85 (95% CI: 1.51-2.27), p < 0.0001. LNs: lymph nodes

Patients who had their tumor removed endoscopically had worse outcomes as compared to those who underwent partial or total gastrectomy with HR = 2.27 (95% CI: 1.14-4.50), as demonstrated in Figure 5. Additionally, those who had partial esophagectomy along with gastric resection had worse survival compared to the patients who only had a partial or total gastrectomy, with HR = 1.42 (95% 1.12-1.80), as also demonstrated in Figure 6. The degree of lymphadenectomy was also an important prognostic factor. Patients who had less than 15 lymph nodes removed at the time of surgery had worse OS as compared to those who had 15 or more lymph nodes removed, with HR = 1.85, (95% 1.51-2.27), p < 0.0001, as demonstrated in Figure 7. Given the limitations associated with chemotherapy and radiotherapy data in the SEER database, they were not included in our survival analyses.

Gender, tumor size, and tumor grade were not predictive of survival on univariate analysis. Therefore, age, race, histologic subtypes, tumor location, surgical approach, and degree of lymphadenectomy were entered in a multivariate Cox proportional hazard ratio model. Only patients who had available results for all parameters were analyzed in this model (n = 1132) as demonstrated by Figure 1. Due to very small numbers (n = 12), non-Hispanic American Indians were excluded from the model.

Results of multivariate analysis

Using the stepwise Cox proportional hazard ratio model, the following factors were predictors for worse survival outcomes: age more than 60 years old with HR = 2.03 (95% CI: 1.49-2.76), patients who had less than 15 lymph nodes examined with HR = 1.72 (95% CI: 1.34-2.20), non-Hispanic whites and non-Hispanic blacks with HR = 1.62 (95% CI: 1.26-2.08), and those who had gastric cancer at the cardia and fundus of the stomach with HR = 1.51 (95% CI: 1.21-1.89).

Prognostic index

**Figure 8:**
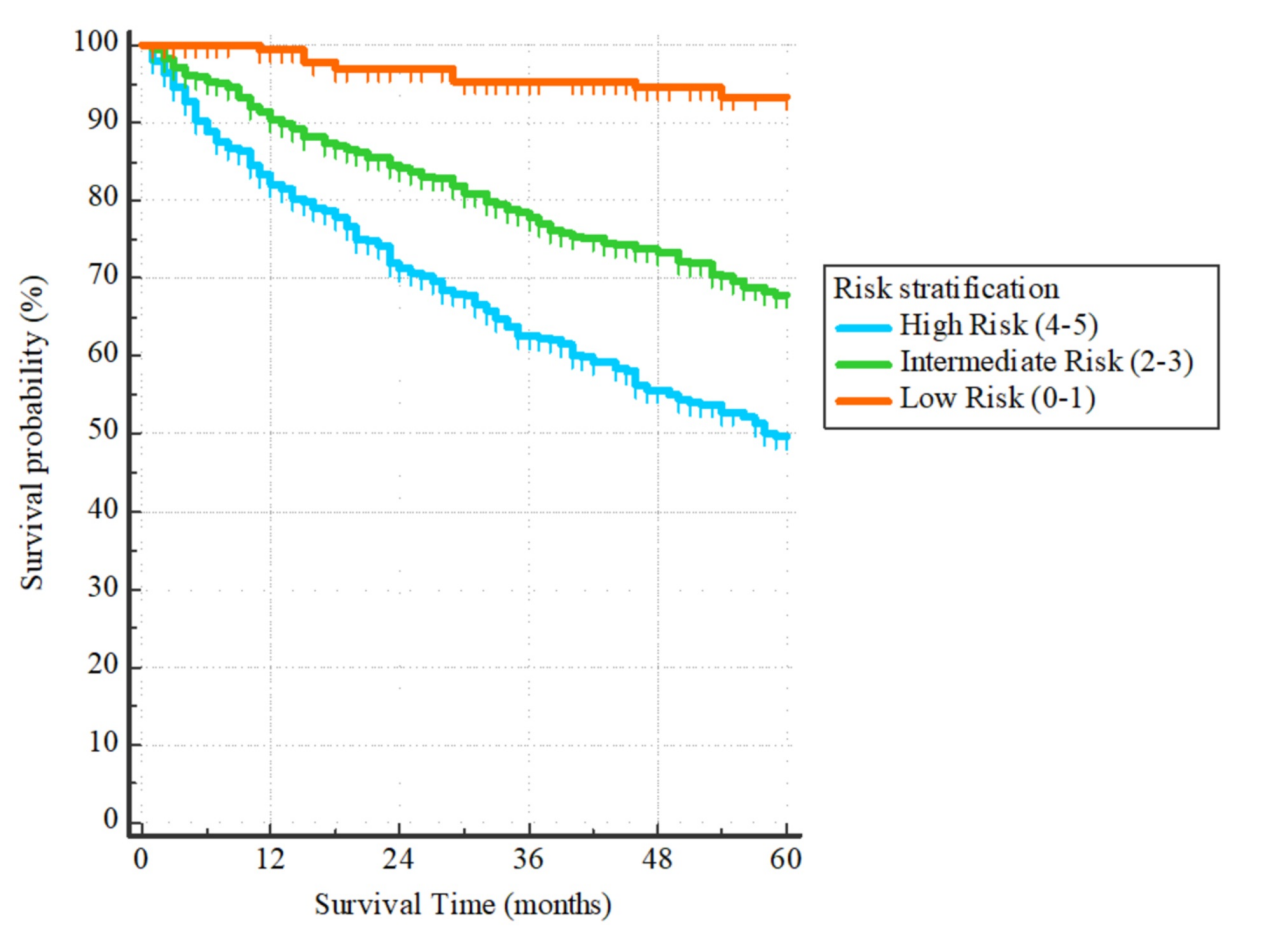
Based on the prognostic score created from the multivariate Cox Regression model, patients were segregated into three risk categories Hazard ratios: high/low = 10.35 (95% CI: 7.53-14.23), high/intermediate = 1.83 (95% CI: 1.45 to 2.31), intermediate/low = 5.65 (95% CI: 4.15-7.71)

Based on the hazard ratios, a risk score was assigned to each of the independent factors determined from the final Cox regression model. The scores were assigned by finding the integer values of the hazard ratios of the corresponding factors. Therefore, a risk score of 2 was assigned to age and 1 to each of the other factors. Finally, the total risk score was determined from the sum of all the four factors (range 0 to 5, Table 2). Three risk categories were determined: low (score 0-1, n = 159), intermediate (score 2-3, n = 510), and high (score 4-5, n = 463). The corresponding five-year OS for the three groups are 93%, 68%, and 50%, respectively (Figure 8). The hazard ratios of death for the high-risk group in comparison to the intermediate and low-risk groups are 10.35 (95% CI: 7.53-14.23) and 1.83 (95% CI: 1.45 to 2.31), respectively. In comparison, the hazard ratio for the intermediate-risk group as compared to the low-risk one is 5.65 (95% CI: 4.15-7.71).

**Table 2:** Results of the final multivariate Cox Regression model and risk scores of independent factors from the 1132 patients who had all parameters available

Covariate	HR	95% CI	P	Risk Score
Age (60 years or older vs <60)	2.03	1.49 to 2.76	<0.0001	2
Race (Non-Hispanic whites or blacks vs Hispanics or Non-Hispanic Asians)	1.62	1.26 to 2.08	0.0001	1
Lymph nodes resected (< 15 vs > 15)	1.72	1.34 to 2.20	<0.0001	1
Primary site (cardia or fundus vs body, antrum or pylorus)	1.51	1.21 to 1.89	0.0003	1

## Discussion

Limited data are available regarding the best treatment option for stage IB gastric cancer, which involves only muscularis propria without lymph nodes involvement. The aim of this study is to identify the adverse prognostic factors in stage IB gastric adenocarcinoma and thus recognize the subjects that could potentially benefit from multimodality treatment. The Intergroup-0116 trial (SWOG 9008) randomized 603 patients with stage IB to IV (M0) gastric adenocarcinoma into surgical resection alone vs surgery with adjuvant chemoradiation in the form of 5-fluorouracil/leucovorin and 45 GY radiation at the locoregional site of the tumor. Adjuvant chemoradiation significantly improved survival in those subjects, However, the number of subjects with stage 1B was unclear. Moreover, only 83 patients had N0 disease. Therefore, it is difficult to draw a conclusion from this study regarding the benefit of adjuvant therapy in stage IB gastric cancer [5].

The Medical Research Council Adjuvant Gastric Infusional Chemotherapy (MAGIC) trial is another landmark trial that randomized 553 patients with operable adenocarcinoma of the stomach (74%), distal esophageal (7%), and esophagogastric (15%) with submucosa involvement and beyond into surgery alone vs surgery with perioperative ECF (epirubicin, cisplatin, and 5-fluorouracil). There was an improvement in the overall survival and progression-free survival and a 25% reduction in the risk of death at five years with the use of perioperative chemotherapy as compared to surgery alone [6]. Tumor stage was determined by either imaging or laparoscopy. Therefore, it is unfeasible to draw a conclusion on the pathologically staged T2/N0 gastric cancer patients in this study.

Seyedin et al. compared surgery alone, surgery with chemotherapy, radiotherapy, or both in non-metastatic gastric adenocarcinoma patients between 1988 and 2008 using the SEER database [7]. They used cause-specific survival as an outcome to compare these modalities. Patients with stage I gastric cancer who were treated with surgery alone had better five-year cause-specific survival as compared to multimodality treatment while patients with stage II and beyond benefited more from multimodality treatment. On the other hand, in their subgroup analysis, patients with stage IB gastric adenocarcinoma who had multimodality treatment had better cause-specific survival compared to those who had surgery alone. Their conclusion should be taken with caution since patients who had multimodality treatment were the second youngest group in their study, which could confound the outcome. Moreover, additional variables that could affect the survival outcome were not considered, such as the number of lymph nodes examined during the surgery and histological subtypes. Furthermore, the SEER database doesn’t provide accurate information regarding the timing of chemotherapy and radiation therapy relative to the surgery. In addition, chemotherapy data are categorized as either “yes - patient had chemotherapy” or “no/unknown - no evidence of chemotherapy was found in the medical records examined.” Finally, although cause-specific survival would provide reliable information about the effectiveness of the treatment, it doesn’t account for the treatment toxicity effect, especially that many patients with gastric cancer are diagnosed in advanced age, which makes them more prone to treatment side effects. Therefore, overall survival would provide more reliable data regarding the effectiveness and the suitability of treatment.

National Comprehensive Cancer Network (NCCN) guidelines do not recommend adjuvant chemotherapy routinely following R0 resection for T2N0M0 gastric adenocarcinoma. However, it is recommended for high-risk patients, those with vascular, lymphatic, and perineural invasion, poorly differentiated or high-grade tumors, and those under the age of 50 [8]. Our analysis showed that age more than 60 years, inadequate lymphadenectomy with less than 15 lymph nodes removed at the time of the surgery, tumor location within the cardia and fundus of the stomach, and non-Hispanic whites and non-Hispanic blacks had a statistically significant worse survival. We were able to create a prognostic score that segregated patients into three risk categories with OS that ranged between 50% and 93%.

The metastatic to non-metastatic lymph nodes ratio has been proposed as an adjunctive tool to the TNM staging system in order to further risk stratify gastric cancer patients and predict their survival by Agnes et al. [9]. Our study showed that inadequate lymphadenectomy is an independent risk factor for poor outcomes. This finding goes in hand with Agnes et al.'s finding and indicates inadvertently down-staged patients with a low number of examined lymph nodes.

Proximal gastric tumors (cardia and fundus of the stomach) were associated with worse survival as compared to distal gastric tumors (body and antrum) with an HR of 1.51 (95% CI 1.21-1.89) on multivariate analysis in our study. Proximal gastric (PG) cancers are usually asymptomatic unless large enough to cause dysphagia. On the other hand, small distal gastric (DG) tumors usually cause disabling heartburn due to gastric juice retention. Also, DG tumors tend to be smaller, being diagnosed at an earlier stage as compared to PG tumors [10]. Moreover, PG tumors tend to harbor aggressive histological subtypes with the capability of early lymphovascular invasion as compared to the distal types due to the difference in their genetic profile [11].

In the univariate analysis, patients who had part of their esophagus removed at the time of surgery or had removal of their tumor endoscopically had a statistically significant worse overall survival. However, the type of surgical resection didn’t affect the overall survival in multivariate analysis. This could be due to inadequate tumor removal in this subgroup. Particularly, our cohort was based on the SEER database between 2004 and 2015, in which endoscopic tumor resection might be suboptimal in these patients. Also, selection bias in this subgroup could explain their poor outcome due to unidentified poor prognostic features.

Regarding the histological subtypes; we categorized the patients according to the World Health Organization (WHO) classification: diffuse, adenocarcinoma NOS, intestinal type, signet ring, and mucinous. Other rare subtypes, including adenocarcinoma with mixed subtypes (n = 30), adenocarcinoma arising from a polyp or an adenoma (n = 21), tubular adenocarcinoma (n = 17), and papillary adenocarcinoma (n = 5), were grouped with adenocarcinoma NOS. Adenocarcinoma NOS was associated with poor survival in the univariate analysis in our study, but this did not hold on multivariate analysis. Given the small number of patients in most of these subtypes, it is difficult to draw a conclusion from our study with regards to histologic subtypes.

Our study revealed that non-Hispanic whites and blacks had worse overall survival as compared to other racial descents. Non-Hispanic whites comprised the most frequent racial descent in our cohort (625 patients, 48%) and 48% of them had PG tumors. On the other hand, only 15% of the Hispanics/non-Hispanic Asians and Pacific Islanders had PG tumors and 10% of non-Hispanic blacks had PG tumors. The proximal tumor location could explain the worse prognosis in the non-Hispanic white group in the univariate analysis. However, the poor survival for both non-Hispanic whites and non-Hispanic blacks as compared to Hispanics, Asian, and Pacific Islanders in a multivariate analysis could point toward the role of certain genetic factors on survival in gastric cancer patients. A recent analysis from a large US population revealed poor H-pylori screening in patients with gastric cancer [12]. H-pylori infection has been implicated in both intestinal and diffuse-type gastric cancer. The interaction between host genetic factors and H-pylori infection could, in part, explain the high gastric cancer mortality in these ethnic groups.

Wang et al. compared gastric cancer-specific survival (GCSS) and overall survival between T1N1M0 and T2aN0M0 gastric cancer (both stage IB) using the SEER database between 2004 and 2015. Their study revealed a clear difference in the survival outcomes between T2N0M0 and T1N1M0 when less than 15 lymph nodes were examined in the surgical specimen in both groups. Moreover, they suggested that stage T2N0M0 wouldn’t benefit from adjuvant treatment in contrast to stage T1N1Mo [13]. In their multivariate analysis for stage 1B gastric cancer (both T1N1M0 and T2N0M0), age more than 70, proximal tumors, and family income less than $60,000 associated with poor GCSS and OS. In our study, we decided to analyze the SEER database for T2N0M0 stage 1B gastric cancer only due to the controversy regarding the best treatment modality for that group. Also, due to the lack of accuracy of the SEER database with regards to chemotherapy and radiation therapy; we excluded using them in our analysis.

Our study has certain limitations. The data were analyzed retrospectively and, therefore, a definitive cause-effect relationship between the variables and the outcome can’t be concluded and additional prospective studies are warranted to validate our results. We included only patients who had surgical resection of the tumor only since surgery is the mainstay of treatment for T2N0M0 gastric cancer. Therefore, our results are applicable only to pathologic rather than clinical staging. Because adenocarcinoma NOS comprises most of the histological subtypes, drawing a meaningful conclusion about their role on survival was limited. Furthermore, certain important potentially confounding variables, such as lymphovascular invasion and perineural invasion, which could potentially affect the outcome of early gastric cancer were not included in our study due to the lack of their availability in the SEER database. Lastly, we did not include data about chemotherapy or radiation therapy due to the inherent inaccuracy of reporting such information in the SEER database [14].

## Conclusions

Patients with a pathological stage T2aN0M0 gastric adenocarcinoma who had inadequate lymph nodes resection (<15 lymph nodes) and whose tumor involves the cardia or the fundus of the stomach have a poor prognosis as compared with their counterparts. Therefore, adjuvant chemotherapy should be considered for these high-risk patients. Prospective cohort studies on patients with gastric adenocarcinoma that is limited to muscularis propria are needed to determine the subgroups who could potentially benefit from adjuvant chemotherapy.
